# Seasonal persistence of faecal indicator organisms in soil following dairy slurry application to land by surface broadcasting and shallow injection

**DOI:** 10.1016/j.jenvman.2016.08.047

**Published:** 2016-12-01

**Authors:** Christopher J. Hodgson, David M. Oliver, Robert D. Fish, Nicholas M. Bulmer, A. Louise Heathwaite, Michael Winter, David R. Chadwick

**Affiliations:** a Rothamsted Research, North Wyke, Okehampton, Devon, EX20 2SB, UK; b Biological & Environmental Sciences, Faculty of Natural Sciences, University of Stirling, Stirling, FK9 4LA, UK; c School of Anthropology & Conservation, University of Kent, Canterbury, Kent, CT2 7NR, UK; d The Lancaster Environment Centre, Lancaster University, Lancaster, LA1 4YQ, UK; e Department of Politics, Amory Building, Rennes Drive, Exeter, Devon EX4 4RJ, UK; f School of Environment, Natural Resources and Geography, Bangor University, Bangor, Gwynedd, LL57 2UW, UK

**Keywords:** Diffuse microbial pollution, *E. coli* die-off, Manure management, Survival curves, Organic fertiliser, Pathogen risk

## Abstract

Dairy farming generates large volumes of liquid manure (slurry), which is ultimately recycled to agricultural land as a valuable source of plant nutrients. Different methods of slurry application to land exist; some spread the slurry to the sward surface whereas others deliver the slurry under the sward and into the soil, thus helping to reduce greenhouse gas (GHG) emissions from agriculture. The aim of this study was to investigate the impact of two slurry application methods (surface broadcast versus shallow injection) on the survival of faecal indicator organisms (FIOs) delivered via dairy slurry to replicated grassland plots across contrasting seasons. A significant increase in FIO persistence (measured by the half-life of *E. coli* and intestinal enterococci) was observed when slurry was applied to grassland via shallow injection, and FIO decay rates were significantly higher for FIOs applied to grassland in spring relative to summer and autumn. Significant differences in the behaviour of *E. coli* and intestinal enterococci over time were also observed, with *E. coli* half-lives influenced more strongly by season of application relative to the intestinal enterococci population. While shallow injection of slurry can reduce agricultural GHG emissions to air it can also prolong the persistence of FIOs in soil, potentially increasing the risk of their subsequent transfer to water. Awareness of (and evidence for) the potential for ‘pollution-swapping’ is critical in order to guard against unintended environmental impacts of agricultural management decisions.

## Highlights

• The method and timing of the slurry applications has an effect on FIO survival.
• FIOs survive longer when applied by shallow injection than by broadcast application.
• FIOs survive longer when applied in October compared to July, or May.
• FIOs die off up to 4 times faster by broadcast application than by shallow injection.

## 1 Introduction

Water used for recreation, drinking or food production (including shell fisheries) is routinely screened for faecal indicator organisms (FIOs) by regulators to track compliance with health related standards and associated legislation (Pachepsky et al., 2016, Clements et al., 2015). The detection of FIOs in environmental matrices is indicative of faecal contamination and their presence in high numbers can suggest a risk to human health in addition to posing wider economic and environmental threats (Oliver et al., 2016a, Oliver et al., 2016b, Quilliam et al., 2015). The Water Framework Directive (WFD), a significant piece of EU water legislation, was designed to protect and improve the quality of water bodies throughout Europe. Microbial pollution of water is integral to the WFD, with bathing and shellfish harvesting waters designated as ‘Protected Areas’ within Article 6 of the WFD. In the USA, total maximum daily loads (TMDLs) are calculated under the Clean Water Act, and FIOs are the leading cause of TMDL exceedance, and thus impairment, of river and stream water quality (USEPA, 2015). In recent years significant international effort has focused on minimising diffuse pollution from agriculture in recognition of the complexity of challenges in mitigating its impact on the water environment (Collins et al., 2016, Brown and Froemke, 2012, McGongle et al., 2012).

In 2013 there were 9.84 million cattle and calves in the UK of which 1.78 million were dairy cattle (Defra, 2014). The majority of these cattle are concentrated in the west of the UK, where grassland agriculture dominates. Approximately 65% of the dairy cattle housing systems are slurry-based systems (Anon, 2006), where the slurry is a mixture of faeces, urine and water. These systems produce *ca*. 50 million tonnes of slurry annually in the UK (Williams et al., 2000). The livestock industry in the UK is intensifying with slurry-based systems being favoured over systems producing solid manures and subsequently the larger animal enterprises are producing greater volumes of slurry. Slurry remains liquid during storage and therefore does not compost, potentially allowing prolonged survival of FIOs and pathogens and opportunities for frequent re-inoculation from recurring inputs to slurry stores (Blaiotta et al., 2016, Hutchison et al., 2005). Animal slurries contain significant concentrations of nutrients, are a desirable farm resource, and are routinely applied to agricultural lands as a crop fertiliser and soil conditioner. However, manure applications can pose a risk for the transfer of pathogenic microorganisms to watercourses via overland flow from fields or from diffuse inputs via artificial drainage or other subsurface hydrological pathways (Cho et al., 2016). The FIO concentrations contained within animal manures are highly variable depending on shedding rates, manure type (liquid or solid manure) and storage conditions. Their rate of decline after manure application to land has been shown to be dependent on environmental factors, such as UV exposure, temperature, soil type and desiccation (Park et al., 2016, Stocker et al., 2015). Agricultural land that receives manure and demonstrates hydrological connectivity to surface waters has the potential to contribute to diffuse microbial pollution of water (Dymond et al., 2016). However, the risk of microbial loss from land to water will vary across contrasting seasons and according to different methods of manure application, and this warrants further investigation.

One hypothesis is that FIOs delivered to injection slots in soil will survive for longer than FIOs in slurry that has been surface broadcast, with potential for prolonging the risk of FIO contamination of the wider environment. This is because of increased cell protection from UV, desiccation and extremes in temperature afforded by the soil habitat. However, an effective method for reducing emissions of ammonia (NH_3_) from the land application of organic manures is to inject slurries into the soil (Häni et al., 2016, Misselbrook et al., 2002). Thus, in many European countries it is the norm to inject slurries below the sward and into the soil, though in the UK slurry application to land via broadcast application, often using a splash plate applicator, remains standard practice. Consequently, there is a need to determine if reducing NH_3_ emissions through shallow injection of slurry will simultaneously increase the potential survival of FIOs, and hence the subsequent risk of increased subsurface transfers of FIOs to water, i.e. the potential for so-called ‘pollution swapping’. The management and mitigation of such risk is becoming a priority for environmental guardians who seek practical tools to facilitate effective microbial risk assessments of agricultural systems (Muirhead, 2015, Oliver et al., 2010, Oliver et al., 2009). The ability of emerging risk-based decision support tools to estimate FIO survival (or rather the accumulation of an FIO burden) in the landscape from a range of on-farm activities, for example the applications of animal slurries to crops and pasture, is important for helping to understand the contribution of diffuse agricultural sources to the impairment of microbial water quality.

Slurry applications to land are influenced by seasons, generally guided by crop requirement, but also by the farmers' need to relieve stress in the capacity of slurry stores prior to housing livestock over the winter. Relatively large volumes of slurries are often applied to agricultural land through the spring, summer and autumn (Smith et al., 2001) and in the UK the Nitrate Pollution Prevention Regulations 2008 (Anon, 2008) have resulted in ∼68% of agricultural land in England being designated as Nitrate Vulnerable Zones (NVZs). NVZs stipulate closed periods during which organic manures with high available nitrogen contents, such as livestock slurries, cannot be applied to land. For grasslands, predominantly livestock farms in the ‘wetter west’ of England, these closed periods extend from 1 September to 31 December on sandy and shallow soils and from 15 October to 15 January on all other soils (Defra, 2013). While much research has focused on nitrate and ammonia emissions from slurry applications to grasslands in NVZs, less attention has been given to microbial pollutants such as FIOs and potential pathogens.

The overall aim of this study was to evaluate the persistence profiles of two key FIOs, *E. coli* and intestinal enterococci (IE), following their delivery to grassland soil through contrasting slurry application methods. The specific objectives were to: (i) determine decay rates and half-lives of *E. coli* and IE at the plot scale following shallow injection and surface broadcast (splash-plate) application of slurry to grassland; and (ii) evaluate whether the resulting FIO die-off patterns in soil were influenced by contrasting season of slurry application in the UK.

## 2 Materials and methods

### 2.1 Site description

Experiments were conducted at the North Wyke Research farm, Devon UK (50°45′N, 3°50′W), on an experimental grassland field. The average annual air temperature of the site is 9.6 °C, and the annual precipitation is 1055 mm (30-year mean, climate record of North Wyke, 1982–2012). The soil type was a poorly drained silty clay loam (Halstow Series; Findlay et al., 1984). Slurry was applied to replicated grassland plots, approximately 2 m × 2 m, using two simulated spreading techniques; surface broadcast (splash-plate) and shallow injection. Broadcast spreading was simulated using an adapted watering can with a spoon attachment to provide a suitable splash-plate spread pattern. In order to simulate the shallow injection, 5–6 cm deep slots were cut into the ground, 20 cm apart, and a watering can was used to pour the appropriate quantity of slurry into the slots to match the same application rate of the surface broadcast slurry. Dairy slurry was obtained from a slurry lagoon on a nearby dairy farm. One day prior to the date of application, slurry was collected from the farm in a 1 m^3^ intermediate bulk container (IBC) and transported to the field. On the morning of application (day 0) the slurry was thoroughly mixed prior to decanting into clean 10 l galvanised steel watering cans for spreading. The slurry was stored in the IBC in the field for the duration of each experiment to enable FIO die-off rates in stored slurry to be determined in addition to the soil-associated FIO die-off rates.

Slurry was applied at the equivalent rate of 45 m^3^ ha^−1^ for all treatments. The plot-scale experiment comprised fifteen 4 m^2^ randomised plots accommodating three treatments (five replicates of; (i) broadcast applied; (ii) shallow injection; and (iii) control plots, no amendments applied) which were investigated during three distinct periods of the year: spring (day 0 = May), summer (day 0 = July) and autumn (day 0 = October). The plots had no history of livestock grazing, manure application or fertiliser addition during the previous 20 years. A different set of fifteen randomised plots was used for each seasonal experiment and a 2 m buffer surrounded each plot to minimise cross contamination of the plots. Meteorological data was collected in the field using a Skye Minimet 4 meteorological station (Skye Instruments Ltd., UK).

### 2.2 Sample collection

The FIO content of the slurry prior to application was determined on day zero of each slurry application (i.e. the day before infield sampling commenced) by removing 5 × 200 ml samples (i.e. 1000 ml) from the IBC. This sample was transported to the laboratory and analysed for FIO concentrations within 2 h.

To determine FIO concentrations in the soil, five cores were taken from each plot, bulked and the soil homogenized. Samples were taken on day 1, 2, 3 and 4 after slurry application, and then weekly with a reduced sampling frequency thereafter until FIO concentrations were undetected or had reached background levels for two consecutive samples. For the control and surface broadcast slurry plots, soil (and overlying slurry) was sampled to a depth of 2 cm using a 2.5 cm diameter auger. However, to facilitate sampling of the shallow injection slurry slots, a 7.5 cm deep auger was used. On average, the slots were 5.5 cm deep and so the 7.5 cm corer effectively retrieved a 2 cm soil sample from the base of the slot. This sampling methodology ensured consistency for all treatments with regard to the volume of soil that was sampled alongside the slurry, thus eliminating any bias from soil dilution on the resulting FIO counts for the deeper 7.5 cm cores. Soil augers were washed and disinfected in a 1% solution of Virkon^®^ and then rinsed three times with sterile deionised water to eliminate cross plot contamination. The slurry in the IBC was mixed and then sampled on every occasion that the plots were sampled for soil cores.

### 2.3 Determination of FIO concentrations

A sub-sample of the slurry (from the IBC), or soil and slurry composite (from the cores), was dried at 105 °C for 24 h to determine the gravimetric water content. A further five grams of target material (either slurry from the IBC or cores of mixed soil and slurry from each plot (n = 5)) was added to sample tubes containing 45 ml of sterile Ringers solution (Oxoid, Basingstoke, UK). Sample tubes were then vortex mixed followed by shaking for 60 min at 150 rpm (Lukham R100 rotatest shaker, LUKHAM ltd., UK) at ambient temperature. After standing for 5 min, 1 ml of the eluent was aseptically transferred to 9 ml of sterile Ringers solution and appropriate serial tenfold dilutions were made. Standard UK Environment Agency methods of membrane filtration were used to determine bacterial concentrations (EA, 2009). Samples were washed through the filtration unit with 20 ml of sterile Ringers solution to aid the dispersion of the bacteria over the entire surface of the membrane filter during the filtering process. Membrane filters of 0.45 μm pore size (Pall Gellman Sciences) were aseptically transferred either to Membrane Lactose Glucuronide Agar (MLGA) (Oxoid) and incubated inverted at 44.5 °C (±0.2 °C) for 18–24 h for *E. coli* or to Slanetz and Bartley agar (Oxoid) and incubated at 37.0 °C (±0.2 °C) for 44–48 h for IE. All FIO concentrations were analysed in the laboratory within 4 h of sample collection. After the total incubation period all plates were examined and all colonies were counted. Initially the counts were reported as presumptive and were subsequently confirmed once further diagnostic studies were undertaken. API^®^ 20E (*E. coli*) and API^®^ 20 Strep (I. E.) biochemical kits (bioMérieux) were used as a confirmatory procedure on ca. 25% of the samples. The API biochemical kits rely on the biochemical profiles exhibited by the bacterial isolates for confirmation of their identity through database comparison.

### 2.4 Statistical analyses

All FIO counts were converted to dry weight equivalents and underwent log_10_ transformation prior to statistical analysis using GenStat (Edition 10.1, Rothamsted Research). Data were analysed either using simple linear regression or using nonlinear regression analysis. For the July and October data the decay rate (*k1/*d) and half-life were determined from the fitted equation A + B*(R**X), where *k1* = -log (R) and half-life = log_e_ (2)/*k1*. For the May data the slope of the regression line is –*k*, where *k* is the first order decay rate on the original scale and half-life = log _e_ (2)/*k*. Multifactorial analysis of variance (ANOVA) and Tukey multiple comparison tests were used to test for differences in decay rates and half-lives between FIO types, application methods and seasons of application. Statistical significance was evaluated at the 0.05 probability level.

## 3 Results

### 3.1 May application

**Fig. 1:**
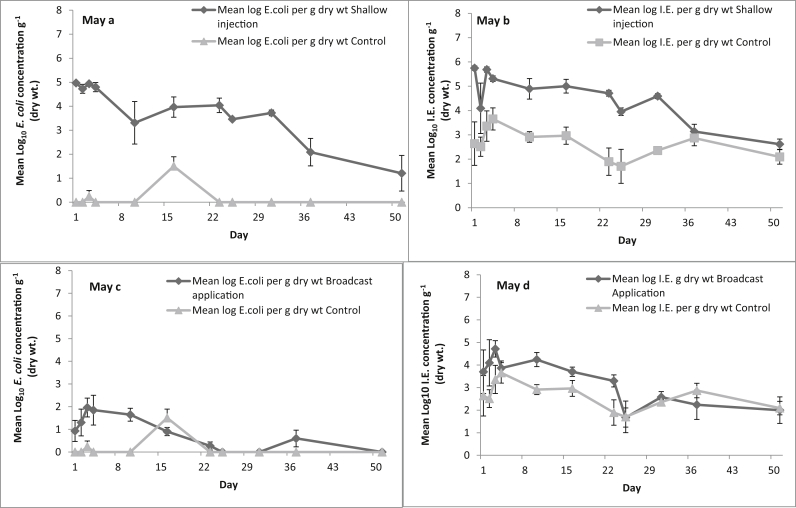
The effect of slurry application method on FIO counts over time in spring (Day 0 = May); Shallow injection, a *E. coli*, b Int. Ent; Surface broadcast, c *E. coli*, d Int. Ent. Data points are the mean of five replicates ± the standard error.

Concentrations of FIOs in the fresh dairy slurry for the May application, reported as colony forming units (CFU), were 6.10 (±0.04) log_10_ and 6.64 (±0.04) log_10_ CFU g^−1^ dry weight for *E. coli* and IE, respectively. A steady decline in concentration of both *E. coli* and IE was evident from the shallow injection plots (Fig. 1a & b). Background concentrations of *E. coli* remained largely below the level of detection (<10 cfu g^−1^) for the 53 days of sampling following the spring application. On the 16th day after application there was a spike in *E. coli* numbers, detected on four out of the five control plots, that coincided with the silage cut of the experimental plots. However, perhaps of greater interest were the concentrations of IE (often at values exceeding 1000 CFU g^−1^) detected in the soil from the control plots. Relative to plots accommodating the shallow injection treatment, the control plots recorded considerable variation in IE counts. While *E. coli* remained above numbers recorded in the control plots beyond day 50, the concentrations of IE declined to within the control plot numbers by day 37. Initial concentrations of *E. coli* detected in the soil from the broadcast applied slurry plots (Fig. 1c & d) were relatively low, although a one-log increase in concentration was detected over the first three days. Numbers of *E. coli* declined to background concentrations in the broadcast treatment by day 23. A similar one-log increase over the first three days with a decline to control concentrations by day 23 was observed with IE. However, background concentrations of IE from the control plots showed no significant difference from the broadcast slurry applied plots. Decay rates (*k*) and half-life for both FIOs were determined (Table 1) for each of the experimental plots (i.e. the five shallow injection and five broadcast slurry applied plots) by fitting a simple linear regression line. The goodness of fit of the regression line was significant (*p* = 0.01) for both FIOs for the shallow injection plots and for IE from the broadcast applied slurry plots. In contrast there was a poor fit to the observed *E. coli* data from the broadcast applied slurry plots, and *p* values ranged from 0.11 to 0.57 (n = 5). For comparison, a two-log decline in viable *E. coli* concentration (from 6.1 to 4.1 log_10_ CFU g^−1^ dry weight) in the slurry taken from the IBC was observed for the 51 days for May. Viable IE concentrations reduced by 0.7 log from 6.5 to 5.8 log_10_ CFU g^−1^ within the slurry store over the 51 days for May.

### 3.2 July application

**Fig. 2:**
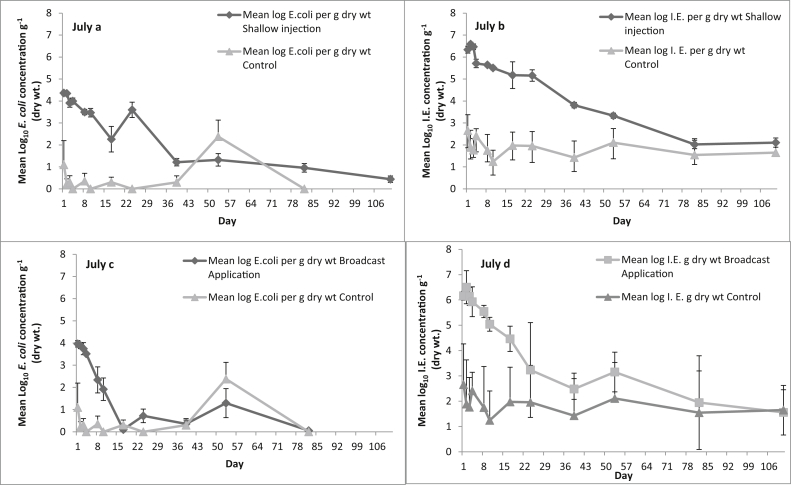
The effect of slurry application method on FIO counts over time in summer (Day 0 = July); Shallow injection, a *E. coli*, b Int. Ent; Surface broadcast, c *E. coli*, d Int. Ent. Data points are the mean of five replicates ± the standard error.

The slurry applied to plots in July had an initial *E. coli* concentration of 5.86 (±0.14) log_10_ CFU g^−1^ dry weight and recorded 6.80 (±0.03) log_10_ CFU g^−1^ dry weight for IE. Fig. 2a & b show the mean FIO concentrations in the soil taken from the injection slots along with the corresponding FIO concentrations enumerated from the soil in the control plots. Both FIOs showed a decline over time, which when fitted to an exponential curve, gave a significant fit to the observed data (*p* < 0.001).

*E. coli* concentrations declined rapidly in the soil to which slurry was broadcast applied, falling to values associated with the control plots on day 17 (Fig. 2c & d). The spike in *E. coli* concentration at day 53, seen in both the broadcast slurry and control plots, coincided with the first rain since day 10. The IE concentrations in the soil to which slurry was broadcast applied declined rapidly to day 24, when their rate of decline slowed, mirroring the concentrations seen in the soil from the control plots. The decline of FIOs from the control plots are best represented by a linear regression (*p* = 0.01) for both FIOs (up to day 17 for *E. coli* and day 24 for IE). A 3 log_10_ CFU g^−1^ reduction of viable *E. coli* concentration (from 5.8 to 2.8 log_10_ CFUg^−1^ dry weight) of the slurry in the IBC was observed up to day 82 and no viable *E. coli* was detected at the end of the sampling period on day 111 for the July application. In contrast, viable IE concentrations fell by 1.25 log_10_ CFU g^−1^ (from 6.55 to 5.25 log_10_ g^−1^) within the slurry store over the 111 sampling days for the July application.

### 3.3 October application

**Fig. 3:**
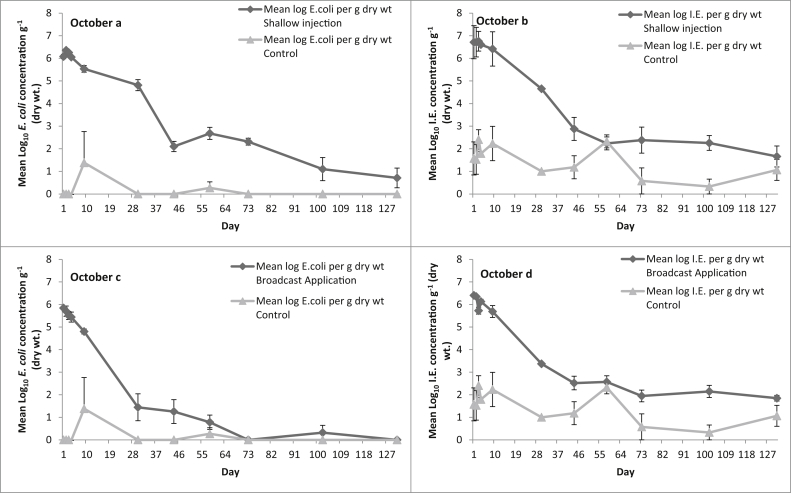
The effect of slurry application on FIO counts over time in autumn (Day 0 = October); Shallow injection, a *E. coli*, b Int. Ent; Surface broadcast, c *E. coli*, d Int. Ent. Data points are the mean of five replicates ± the standard error.

FIO concentrations in the fresh dairy slurry for the autumn application were 6.15 (±0.06) log_10_ and 7.12 (±0.07) log_10_ CFU g^−1^ dry weight for *E. coli* and IE, respectively. Fig. 3a– d show the mean FIO concentrations in the soil taken from the injection and broadcast applied slurry plots along with the corresponding FIO concentrations quantified in the soil of the control plots. Again the decline for *E. coli* and IE in the soil from the shallow injected and the broadcast applied slurry plots is best described by an exponential curve, with a significant fit to the observed data (*p* < 0.001). Concentrations of *E. coli* were detected in the soil from the injection slots up to day 131 but no viable *E. coli* was detected in the soil from the control plots. A relatively rapid die-off was observed for *E. coli* numbers in the soil from the broadcast applied slurry plots over the first 30 days post application, after which this rate of decline slowed. *E. coli* was readily cultured in the soil from the broadcast applied plots for up to 102 days post slurry application. A 2.6 log_10_ CFU g^−1^ decline in viable *E. coli* concentration (from 6.15 to 3.55 log_10_ g^−1^ dry weight) in the slurry contained in the IBC was observed over the 131 sampling days for the October application. In contrast, a 1.2 log_10_ CFU g^−1^ reduction in viable IE concentrations was recorded over the same period (reducing from 7.0 to 5.8 log_10_ CFU g^−1^).

### 3.4 Comparison of FIO decay rates and half lives

FIOs in dairy cattle slurry survived significantly longer when applied to land via shallow injection than by broadcast application (*p* < 0.05). Decay rates of FIOs were up to 4 times quicker when delivered to soil via broadcast application rather than by shallow injection.

*Half-lives*: Multifactorial ANOVA identified that there were significant differences in half-lives between FIO types, between different seasons and between slurry application methods (*p* < 0.05). The half-life of *E. coli* once applied to grassland was significantly shorter than that of IE (*p* < 0.01). In general, FIOs were shown to accommodate a significantly shorter half-life when applied to soil via a broadcast application (*p* < 0.05) and if applied during the spring (*p* < 0.05). A significant interaction was observed between FIO type and season of application (*p* < 0.05), with *E. coli* half-lives influenced by season more strongly than IE.

*Decay rates:* Significant differences in decay rates between FIO types and between seasons were also recorded (*p* < 0.05) but not between application methods (*p* = 0.053), which fell just outside the defined 5% significance level. The significantly slower decay rate was associated with IE (complementing the half-life data) and the highest rate of decay was associated with FIOs applied to land in spring, with summer and autumn both accommodating significantly lower decay rates (*p* < 0.05).

The mean decay rates (*k*) day^−1^ and half-life (*t*_*1/2*_) days for *E. coli* and IE for the five shallow injection and five broadcast slurry applied plots for the May, July and October applications are summarised in Table 1. Mean and maximum measured levels of UV, air temperature and total rainfall and the percentage dry matter for each slurry application are recorded in Table 2.

## 4 Discussion

Plot studies are essential to help consolidate understanding and scale up findings from laboratory-based investigations to field and catchment scales (Winter et al., 2011). It is therefore critically important that plot scale studies of FIO persistence are undertaken to complement observations made at smaller scales and to provide evidence of impacts of complex interacting environmental factors on FIO survival (Oliver et al., 2016a). However, the current evidence-base of FIO persistence patterns delivered to soils through manure applications is currently limited (Stocker et al., 2015). The research reported here contributes important data to help support our understanding of FIO persistence under field-relevant conditions. Our results have highlighted significant impacts associated with both method and timing of slurry applications on the persistence of FIOs in grassland soil across different seasonal conditions in the UK.

Differences were observed in the survival characteristics of the two FIOs under investigation. The population of IE within the slurry was found to be more robust and accommodated a longer half-life and slower decay rate relative to the *E. coli* population. Interestingly, the persistence patterns of *E. coli* and enterococci in soils following manure application (and simulated rainfall) in a US study were also shown to differ but, in contrast to out study, *E. coli* survived better than enterococci (Stocker et al., 2015). Others have also found *E. coli* to out survive enterococci when bovine manure was incorporated into soil (Lau and Ingham, 2001). The latter study held mixed soil and manure treatments under a controlled temperature regime using a laboratory set-up and so the FIOs were not exposed to the same number of interacting and variable environmental factors that challenge FIO survival in the field, which might explain the contradictory findings between their study and ours. Differences in FIO survival between field-based studies are likely to reflect the different environmental conditions specific to each experiment. Our study used a poorly drained silty clay loam soil whereas Stocker et al. (2015) investigated FIO survival in soil of a sandy loam texture. Others have reported *E. coli* and enterococci survival in pig slurry applied to different soil types and found both FIOs to accommodate different survival profiles depending on soil type (Cools et al., 2001). Beyond soil type, factors such as soil moisture, manure type, manure characteristics such as percentage of dry matter and solids, rainfall re-wetting events and soil nutrient status are known to impact on FIO survival profiles (Park et al., 2016, Yao et al., 2015, Bech et al., 2014) and may help explain the differences in reported FIO characteristics between studies.

The timing of manure application to land is clearly important. It is known that manure and slurry applications that coincide with wet weather can help to promote elevated FIO concentrations being transferred from land to water (Blaustein et al., 2016). However, our results also demonstrate that the season within which slurry is applied to land can impact on the survival dynamics of FIOs delivered via both broadcast spreading and surface injection. *E. coli* survived in the soil at the base of the injection slots, albeit at relatively low concentrations, for more than 100 days for both the July and October applications. This contrasts with the May application where *E. coli* was not detected beyond 50 days in the injection slots. Similarly, the *E. coli* population in the dairy cattle slurry applied via broadcast application in the spring declined relatively quickly and was no longer a substantial source of *E. coli* after only 10 days. Therefore, FIO survival was shortest in the warmer spring period of study relative to summer and autumn monitoring, complementing findings that have shown temperature to be an important driver of FIO decline in land-applied manures and slurries (Plachá et al., 2001). It is important to note that mean UV and temperature values for spring were higher than those observed during the summer and so a degree of caution is needed in assuming that we can apply specific decay rates to particular seasons given the potential for variable meteorological conditions (i.e. atypical seasons). Certainly, temperature and soil moisture status have been shown to be significant factors affecting the dynamics of FIOs once introduced into the soil environment (Hutchison et al., 2004) though the exact nature of temperature influences on *E. coli* die-off in agricultural environments have yet to be fully characterised (Martinez et al., 2013). In NVZs of Europe, the closed period for slurry applications extends through autumn to late winter. Given that FIOs introduced to grassland via slurry application in autumn persist significantly longer relative to spring, it is clear that the autumnal ‘closed period’ for slurry applications in the UK delivers multiple benefits; it not only limits nitrate leaching from land to water but also restricts slurry applications when FIOs would persist longest.

The half-lives of both *E. coli* and enterococci were found to be significantly shorter when slurry was applied to grassland plots via broadcast applications to the field surface rather than applied by shallow injection into the soil. Surface applied manures and slurries will be exposed to UV and will likely experience accelerated desiccation due to more rapid warming and drying from wind. Further, unlike cells delivered to soil by surface injection methods, surface applied FIOs will ultimately suffer from lack of a protective niche afforded by the soil matrix and so be more prone to detrimental effects of temperature fluctuations (Avery et al., 2004, Hutchison et al., 2004). A recent meta-analysis of 151 FIO survival datasets revealed significantly faster decay and significantly larger sensitivities in the decline of FIO populations associated with cells delivered to the soil surface compared to those that were mixed or ploughed into the soil matrix (Park et al., 2016). The results from our study are therefore consistent with observations from other areas of the world where similar research has been undertaken.

Interestingly, despite the rapid decay of FIOs observed for spring, an initial short-lived increase in *E. coli* population growth of one-order of magnitude was observed in slurry delivered via the surface broadcast method during the first four days post-application. Admittedly, the starting concentration of the *E. coli* population was very low, and perhaps an influx of cells had transferred into the underlying soil following mobilisation from the overlying slurry during rainfall (Blaustein et al., 2015) rather than this increase representing replication of cells, though it is difficult to say with certainty which is the most likely cause. However, a number of studies of FIO persistence in various faecal matrices have reported on the potential for FIO growth. For example, *Escherichia coli* in bovine manure incorporated into the soil under controlled laboratory-based conditions exhibited a growth phase of ∼1–2 log_10_ CFU g^−1^ over a period of approximately one week, and enterococci numbers were observed to increase too, though to a lesser extent (Lau and Ingham, 2001). Investigations of FIO persistence in cowpats held under field-relevant conditions have also reported on the potential for *E. coli* growth in the immediate period post defecation with increases in population size of ∼1.5 order of magnitude increase over a similar period (e.g. van Kessel et al., 2007, Sinton et al., 2007, Martinez et al., 2013). It may be that in conditions where a significant decline in bacterial population would normally be expected (e.g. elevated temperatures and UV levels) the warming of slurry beneath the immediate crust is sufficient to promote some cell growth, and perhaps to levels that counteract the proportion of cells lost through the process of desiccation in the overlying crust. Conversely, the spikes in FIO populations observed at later stages of the experiment were likely a result of rehydration of the soil matrix following rainfall rewetting of soil and subsequent redistribution of nutrients to the cells (Jamieson et al., 2002).

The abatement of NH_3_ emissions from the livestock sector across Europe has been a focus of research for some time. Emissions of NH_3_ from agriculture can be reduced by an average of 73% when slurry is applied to land by shallow injection in comparison to conventional splash-plate application (Misselbrook et al., 2002). Thus an abatement strategy to reduce NH_3_ emissions from slurry is to shallow inject it into the soil. However, as this current study shows, injecting dairy slurry significantly increases the survival of FIOs in grassland environments compared to broadcast application, potentially sustaining a longer-lasting threat to surrounding water quality if these faecal sources are mobilised and transferred by subsequent rainfall run-off into artificial subsurface drainage systems, known to be efficient conduits of FIO transfer (Oliver et al., 2005). Indeed, some research has highlighted vulnerability of FIO transfers following slurry injection (Amin et al., 2014) and others have suggested that injection actually increases the leaching potential of nitrate, phosphorus and pathogens (Fangueiro et al., 2014). Nevertheless, slurry injection of FIOs into the soil is likely to reduce the risk of ‘incidental’ rapid overland flow losses from land to water following heavy rainfall because the slurry is better protected from detachment mechanisms such as raindrop impact on the soil surface.

## 5 Conclusion

FIOs that are recycled to grassland soils via slurry survive better when applied by shallow injection rather than surface broadcast methods, and decay rates of both *E. coli* and IE vary depending on time of application, likely due to environmental drivers such as temperature and UV, and rainfall effects on soil moisture status. While slurry injection is promoted as a technique to reduce agricultural NH_3_ emissions to the atmosphere it is important to recognise the potential for multi-pollutant impacts from on-farm management decisions. The opportunity for inadvertent FIO transfer from land to receiving waters following shallow injection of slurry is likely to depend on site specific factors such as the presence and depth of tile drains and the soil type and presence of, for example, preferential pathways such as soil macropores. The nuances of potential environmental impacts that might arise following different manure application methods to grassland systems therefore create a complex conundrum for farmers, land-owners and environmental managers who are tasked with delivering on multiple environmental objectives such as balancing reduced odour and atmospheric NH_3_ pollution against potential risks to soil and water quality. The implications of extended FIO survival in soil following slurry application by shallow injection must be addressed in the wider context of ‘pollution swapping’ when designing mitigation strategies for multiple pollutants at the farm scale. In response, larger and longer-term field trials offer scope for improving our understanding of complex multi-pollutant interactions associated with manure and livestock management in agricultural systems.

## Figures and Tables

**Table 1:** Mean decay rates (*k)* and half-life (*t*_*1/2*_) for *E.coli* and intestinal enterococci for data for the experimental plots; mean of five shallow injection (S/I) and five broadcast (B/C) slurry applied plots for the May, July and October applications.

		*E. coli*		Intestinal enterococci	
		*k (day*^*−1*^*)*	t_1/2_ (days)	*k (day*^*−1*^*)*	t_1/2_ (days)
**May**					
S/I	n = 5	0.11	9.68	0.021	36.93
B/C	n = 5	0.23	6.40	0.057	19.61
**July**					
S/I	n = 5	0.023	31.29	0.018	48.33
B/C	n = 5	0.097	9.38	0.042	17.27
**October**					
S/I	n = 5	0.029	34.14	0.025	27.79
B/C	n = 5	0.036	24.14	0.033	27.11

**Table 2:** Mean meteorological data; UV, air temperature and total rainfall for each of three slurry application periods.

Month of application	Sampling period days	Mean, 24 h/Max h UV (wm^2^)	Mean, 24 h/Max h air temp (°C)	Total rainfall (mm)	Slurry dry matter at application (%)
May	51	236.1/836	15.1/26.4	39.0	7.4
July	111	129.6/808	14.9/29.7	25.4	5.9
October	131	60.9/503.5	10.7/17.9	68.4	5.3

## References

[bib1] Amin M.M., Šimůnek J., Lægdsmand M. (2014). Simulation of the redistribution and fate of contaminants from soil-injected animal slurry. Ag. Water Manag..

[bib2] Anon (2006). Farm Practice Survey 2006-England.

[bib3] Anon (2008). The Nitrate Pollution Prevention Regulations SI 2349 September 2008.

[bib4] Avery L.M., Hill P., Killham K., Jones D.L. (2004). *Escherichia coli* O157 survival following the surface and sub surface application of human pathogen contaminated organic waste to soil. Soil Biol. Biochem..

[bib5] Bech T.B., Rosenbom A.E., Kjaer J., Amin M.M., Olsen P., Jacobsen C.S. (2014). Factors influencing the survival and leaching of tetracycline-resistant bacteria and Escherichia coli through structured agricultural fields. Agric. Ecosys. Env..

[bib6] Blaiotta G., Di Cerbo A., Murru N., Coppola R., Aponte M. (2016). Persistence of bacterial indicators and zoonotic pathogens in contaminated cattle wastes. BMC Microbiol..

[bib7] Blaustein R.A., Hill R.L., Micallef S.A., Shelton D.R., Pachepsky Y.A. (2016). Rainfall intensity effects on removal of fecal indicator bacteria from solid dairy manure applied over grass-covered soil. Sci. Total Env..

[bib8] Blaustein R.A., Pachepsky Y.A., Shelton D.R., Hill R.L. (2015). Release and removal of microorganisms from land-deposited animal waste and animal manures: a review of data and models. J. Env. Qual..

[bib9] Brown T.C., Froemke P. (2012). Nationwide assessment of nonpoint source threats to water quality. Biosci..

[bib10] Cho K.H., Pachepsky Y.A., Oliver D.M., Muirhead R.W., Park Y., Quilliam R.S., Shelton D.R. (2016). Modeling fate and transport of fecally-derived microorganisms at the watershed scale: state of the science and future opportunities. Water Res..

[bib11] Clements K., Quilliam R.S., Jones D.L., Wilson J., Malham S.K. (2015). Spatial and temporal heterogeneity of bacteria across an intertidal shellfish bed: implications for regulatory monitoring of faecal indicator organisms. Sci. Total Env..

[bib12] Collins A.L., Zhang Y.S., Winter M., Inman A., Jones J.I., Johnes P.J., Cleasby W., Vrain E., Lovett A., Noble L. (2016). Tackling agricultural diffuse pollution: what might uptake of farmer-preferred measures deliver for emissions to water and air?. Sci. Total Environ..

[bib13] Cools D., Merckx R., Vlassak K., Verhaegen J. (2001). Survival of *E. coli* and Enterococcus spp. derived from pig slurry in soils of different texture. Appl. Soil Ecol..

[bib14] Defra (2014). Agriculture in the United Kingdom 2013.

[bib15] Defra (2013). Guidance for Farmers in Nitrate Vulnerable Zones. https://www.gov.uk/government/uploads/system/uploads/attachment_data/file/432141/pb14050-nvz-guidance.pdf.

[bib16] Dymond J.R., Serezat D., Ausseil A.G.E., Muirhead R.W. (2016). Mapping of *Escherichia coli* sources connected to waterways in the Ruamahanga catchment, New Zealand. Environ. Sci. Technol..

[bib17] Environment Agency (2009). The Microbiology of Drinking Water Part 4—methods for the Isolation and Enumeration of Coliform Bacteria and *Escherichia coli* (Including *E. coli* O157:H7).

[bib18] Fangueiro D., Surgy S., Napier V., Menaia J., Vasconcelos E., Coutinho J. (2014). Impact of slurry management strategies on potential leaching of nutrients and pathogens in a sandy soil amended with cattle slurry. J. Env. Manag..

[bib19] Findlay D.C., Colborne G.J.N., Cope D.W. (1984). Title: soils and their use in south west England. Soil Surv. Bull. Issue.

[bib20] Häni C., Sintermann J., Kupper T., Jocher M., Neftel A. (2016). Ammonia emission after slurry application to grassland in Switzerland. Atmos. Env..

[bib21] Hutchison M.L., Walters L.D., Moore A., Crookes K.M., Avery S.M. (2004). Effect of length of time before incorporation on survival of pathogenic bacteria present in livestock waste applied to agricultural soil. Appl. Env. Microbiol..

[bib22] Hutchison M.L., Walters L.D., Moore A., Crookes K.M., Avery S.M. (2005). Declines of zoonotic agents in liquid livestock wastes stored in batches on-farm. J. Appl. Microbiol..

[bib23] Jamieson R.C., Gordon R.J., Sharples K.E., Stratton G.W., Madani A. (2002). Movement and persistence of fecal bacteria in agricultural soils and subsurface drainage water: a review. Can. Biosyst. Eng..

[bib24] Lau M.M., Ingham S.C. (2001). Survival of faecal indicator bacteria in bovine manure incorporated into soil. Lett. Appl. Microbiol..

[bib25] Martinez G., Pachepsky Y.A., Shelton D.R., Whelan G., Zepp R., Molina M., Panhorst K. (2013). Using the Q10 model to simulate *E. coli* survival in cowpats on grazing lands. Env. Int..

[bib26] McGongle D.F., Harris R.C., McCamphill C., Kirk S., Dils R., MacDonald J., Bailey S. (2012). Towards a more strategic approach to research to support catchment-based policy approaches to mitigate agricultural water pollution: a UK case-study. Env. Sci. Policy.

[bib27] Misselbrook T.H., Smith K.A., Johnson R.A., Pain B.F. (2002). Slurry application techniques to reduce ammonia emissions: results of some UK field-scale experiments. Biosys. Eng..

[bib28] Muirhead R. (2015). A farm-scale risk-index for reducing fecal contamination of surface waters. J. Env. Qual..

[bib29] Oliver D.M., Heathwaite L., Haygarth P.M., Clegg C.D. (2005). Transfer of *Escherichia coli* to water from drained and undrained grassland after grazing. J. Env. Qual..

[bib30] Oliver D.M., Page T., Hodgson C.J., Heathwaite A.L., Chadwick D.R., Fish R.D., Winter M. (2010). Development and testing of a risk indexing framework to determine field-scale critical source areas of faecal bacteria on grassland. Env. Modell. Softw..

[bib31] Oliver D.M., Fish R.D., Hodgson C.J., Heathwaite A.L., Chadwick D.R., Winter M. (2009). A cross-disciplinary toolkit to assess the risk of faecal indicator loss from grassland farm systems to surface waters. Ag. Ecosys. Environ..

[bib32] Oliver D.M., Porter K.D., Pachepsky Y.A., Muirhead R.W., Reaney S.M., Coffey R., Kay D., Milledge D.G., Hong E., Anthony S.G., Page T. (2016). Predicting microbial water quality with models: over-arching questions for managing risk in agricultural catchments. Sci. Total Env..

[bib33] Oliver D.M., Hanley N.D., van Niekerk M., Kay D., Heathwaite A.L., Rabinovici S.J., Kinzelman J.L., Fleming L.E., Porter J., Shaikh S., Fish R. (2016). Molecular tools for bathing water assessment in Europe: balancing social science research with a rapidly developing environmental science evidence-base. Ambio.

[bib34] Pachepsky Y., Shelton D., Dorner S., Whelan G. (2016). Can *E. coli* or thermotolerant coliform concentrations predict pathogen presence or prevalence in irrigation waters?. Crit. Rev. Microbiol..

[bib35] Park Y., Pachepsky Y., Shelton D., Jeong J., Whelan G. (2016). Survival of manure-borne and fecal coliforms in soil: temperature dependence as affected by site-specific factors. J. Env. Qual..

[bib36] Plachá I., Venglovský J., Sasáková N., Svoboda I.A. (2001). The effect of summer and winter seasons on the survival of Salmonella typhimurium and indicator micro-organisms during the storage of solid fraction of pig slurry. J. Appl. Microbiol..

[bib37] Quilliam R.S., Kinzelman J., Brunner J., Oliver D.M. (2015). Resolving conflicts in public health protection and ecosystem service provision at designated bathing waters. J. Env. Manag..

[bib38] Sinton L.W., Braithwaite R.R., Hall C.H., Mackenzie M.L. (2007). Survival of indicator and pathogenic bacteria in bovine feces on pasture. Appl. Env. Microbiol..

[bib39] Smith K.A., Brewer A.J., Crabb J., Dauven A. (2001). A survey of the production and use of animal manures in England and Wales. III. Cattle manures. Soil Use Manag..

[bib40] Stocker M.D., Pachepsky Y.A., Hill R.L., Shelton D.R. (2015). Depth-dependent survival of *Escherichia coli* and enterococci in soil after manure application and simulated rainfall. Appl. Env. Microbiol..

[bib41] USEPA (2015). http://iaspub.epa.gov/waters10/attains_nation_cy.control?p_report_type=T#causes_303d. Accessed on 5.08.16.

[bib42] van Kessel J.S., Pachepsky Y.A., Shelton D.R., Karns J.S. (2007). Survival of *Escherichia coli* in cowpats in pasture and in laboratory conditions. J. Appl. Microbiol..

[bib43] Williams J.R., Chambers B.J., Smith K.A., Ellis S., Petchey T., D'Arcy B., Frost A. (2000). Farm manure land application strategies to conserve nitrogen within farming systems. Agriculture and Waste: Management for a Sustainable Future.

[bib44] Winter M., Oliver D.M., Fish R., Heathwaite A.L., Chadwick D., Hodgson C. (2011). Catchments, sub-catchments and private spaces: scale and process in managing microbial pollution from source to sea. Env. Sci. Policy.

[bib45] Yao Z., Yang L., Wang H., Wu J., Xu J. (2015). Fate of *Escherichia coli* O157: H7 in agricultural soils amended with different organic fertilizers. J. Haz. Mat..

